# Nationwide evaluation of malaria infections, morbidity, mortality, and coverage of malaria control interventions in Madagascar

**DOI:** 10.1186/1475-2875-13-465

**Published:** 2014-11-28

**Authors:** Thomas Kesteman, Milijaona Randrianarivelojosia, Chiarella Mattern, Emma Raboanary, Dolorès Pourette, Florian Girond, Vaomalala Raharimanga, Laurence Randrianasolo, Patrice Piola, Christophe Rogier

**Affiliations:** Malaria Research Unit, Institut Pasteur de Madagascar, BP 1274, Avaradoha, Antananarivo 101 Madagascar; Unité de recherche sur les maladies infectieuses et tropicales émergentes (URMITE), UMR 6236, 27 Boulevard Jean Moulin, 13385 Marseille, Cedex 05, France; Epidemiology Unit, Institut Pasteur de Madagascar, BP 1274, Avaradoha, Antananarivo 101 Madagascar; Centre population et développement (CEPED), Institut de Recherche pour le Développement, 19 Rue Jacob, 75006 Paris, France; Université Catholique de Madagascar, Antananarivo, Madagascar; Institute for Biomedical Research of the French Armed Forces (IRBA), BP 73, 91223 Brétigny-Sur-Orge, Cedex, France

**Keywords:** Malaria, Mortality, Morbidity, Prevalence, Prevention and control, Cross-sectional studies, Health surveys, Vector control, Insecticide-treated bed nets, Case management

## Abstract

**Background:**

In the last decade, an important scale-up was observed in malaria control interventions. Madagascar entered the process for pre-elimination in 2007. Policy making needs operational indicators, but also indicators about effectiveness and impact of malaria control interventions (MCI). This study is aimed at providing data about malaria infection, morbidity, and mortality, and MCI in Madagascar.

**Methods:**

Two nationwide surveys were simultaneously conducted in 2012–2013 in Madagascar: a study about non-complicated clinical malaria cases in 31 sentinel health facilities, and a cross-sectional survey (CSS) in 62 sites. The CSS encompassed interviews, collection of biological samples and verbal autopsies (VA). Data from CSS were weighted for age, sex, malaria transmission pattern, and population density. VA data were processed with InterVA-4 software.

**Results:**

CSS included 15,746 individuals of all ages. Parasite rate (PR) as measured by rapid diagnostic tests was 3.1%, and was significantly higher in five to 19 year olds, in males, poorer socio-economic status (SES) quintiles and rural areas. Long-lasting insecticidal nets (LLIN) use was 41.7% and was significantly lower in five to 19 year olds, males and wealthier SES quintiles. Proportion of persons covered by indoor residual spraying (IRS) was 66.8% in targeted zones. Proportion of persons using other insecticides than IRS was 22.8%. Coverage of intermittent preventive treatment during pregnancy was 21.5%. Exposure to information, education and communication messages about malaria was significantly higher in wealthier SES for all media but information meetings. The proportion of fever case managements considered as appropriate with regard to malaria was 15.8%. Malaria was attributed as the cause of death in 14.0% of 86 VA, and 50% of these deaths involved persons above the age of five years. The clinical case study included 818 cases of which people above the age of five accounted for 79.7%. In targeted zones, coverage of LLIN and IRS were lower in clinical cases than in general population.

**Conclusions:**

This study provides valuable data for the evaluation of effectiveness and factors affecting MCI. MCI and evaluation surveys should consider the whole population and not only focus on under-fives and pregnant women in pre-elimination or elimination strategies.

**Electronic supplementary material:**

The online version of this article (doi:10.1186/1475-2875-13-465) contains supplementary material, which is available to authorized users.

## Background

Malaria control was marked in the last decade by a steep and almost constant increase in international funding. Domestic funding for malaria control increased as well in the same period, although less sharply. The successes of control programmes resulting from availability of resources have led some countries towards pre-elimination, including Madagascar in 2007 [1]. Unfortunately resources available for malaria control are reaching a plateau, especially in low-income countries that depend heavily on international funding [2]. Those countries also bear the highest burden of malaria [3]. In order for malaria control programmes to reduce the impact of this lethal disease, two major options exist: i) a new control tool that performs better at lower cost, or ii) ensuring that interventions deployed perform accurately. To evaluate such performance, individual effectiveness, coverage and community effectiveness need to be monitored [4]. In 2012–2013, a project aimed at measuring the coverage and effectiveness of malaria control interventions in Madagascar in terms of infection, morbidity and mortality, and the factors associated with their effectiveness as measured by both quantitative and qualitative approaches was set up. This project was named MEDALI (an acronym for *Mission d’Etude des Déterminants de l’Accès aux Méthodes de Lutte antipaludique et de leur Impact*), and took place in Madagascar. Herein are presented i) the design and methodology of the quantitative surveys of the MEDALI project; ii) a nationwide assessment of malaria infections, morbidity and mortality; and, iii) an evaluation of the coverage of malaria control interventions in all population groups.

## Methods

A nationwide survey in 2012–2013 in 31 zones representing all transmission patterns of Madagascar was conducted. This survey included a cross-sectional study and a concurrent study about clinical cases. Clinical data, sociodemographic data and exposure to malaria control interventions have been collected.

### Interventions studied

In Madagascar, malaria control relies on the following interventions: i) long-lasting insecticidal nets (LLIN) distribution; ii) indoor residual spraying (IRS) campaigns; iii) intermittent preventive treatment of pregnant women (IPTp); iv) testing of fever cases by rapid diagnostic tests (RDT) or microscopy; v) treatment of non-complicated malaria cases by artemisinin-based combination therapy (ACT); and, vi) information, education and communication (IEC) campaigns.

IPTp was introduced in 2004. In 2006, the national policy switched from chloroquine to ACT as treatment for uncomplicated malaria, together with the use of RDT. Artesunate/amodiaquine combination therapy is the first-line treatment, while artemether/lumefantrine is the second-line treatment with oral quinine as the alternative. In 2008, universal coverage with LLINs was scaled up at national level. While there was a half-century long history of IRS in Madagascar using dichlorodiphenyltrichloroethane (DDT), IRS with pyrethroids was deployed in 2008 in the Central Highlands and Fringe transmission patterns [5]. In 2009–2010, IRS was extended to some South and Western districts through the Global Fund to Fight AIDS, Tuberculosis and Malaria and the US President’s Malaria Initiative; while it was down-scaled to focalized IRS in the Central Highlands. Similarly to other interventions, IEC campaigns were reinforced in the 2000s [6].

### Selection of study zones

**Figure 1:**
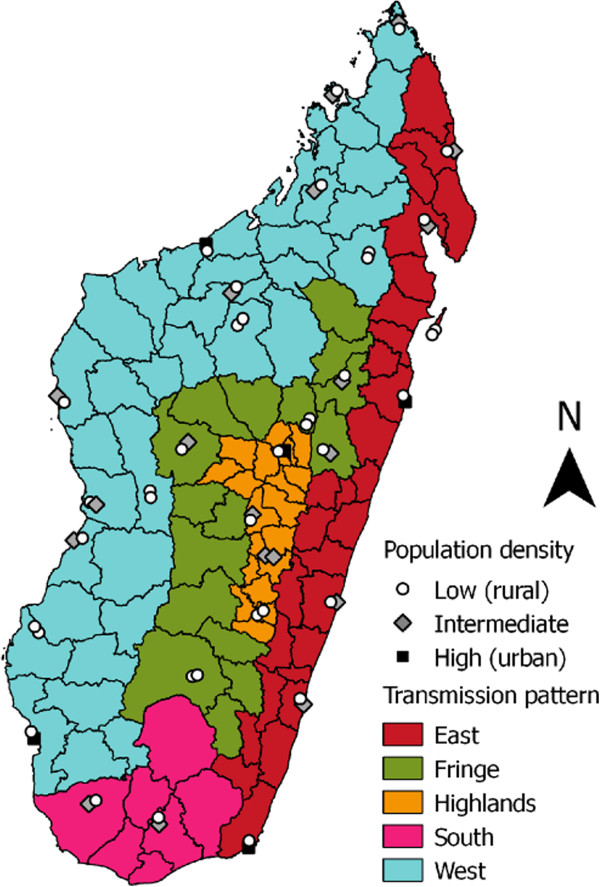
**Malaria transmission patterns in the districts of Madagascar, study sites and their population density.**

Districts of Madagascar are divided in five main operational zones that correspond to the transmission patterns of Madagascar (Figure 1) [7]. The two coastal regions exhibit highest endemic patterns with a transmission lasting all year long in the East and more than six months per year in the West. In the central highlands, the transmission is unstable, and episodic or epidemic. In the Fringe areas, i.e., at intermediate altitudes, the transmission pattern is seasonal, lasting from November to May (rainy season). In the South, the period of transmission is short and episodic. Fringe, Central Highlands and the South are prone to outbreaks.

The selection of study zones was based on a network of sentinel health centres (SHC) that were established in order to cover all the ecosystems of Madagascar for surveillance of fever-associated diseases [8]. Each location, where at least one SHC existed in 2012, was included in the study design defined 31 study zones. All malaria transmission patterns were represented: 13 study zones were located in the Western transmission pattern, seven in the East, five in the Fringe, four in the Central Highlands and two in the South (Figure 1). These patterns encompass, respectively, 21.0, 27.5, 13.7, 31.9, and 5.9% of Malagasy population.

### Cross-sectional survey

The cross-sectional survey (CSS) aimed at studying the prevalence rates of malaria infection and exposure to factors that could be related to malaria, including malaria control interventions. The sample is made of clusters, stratified by transmission patterns.

#### Selection of study sites

For each study zone, two study sites, were defined to make a total of 62 study sites. To achieve this, two villages or *fokontany*, the smallest administrative subdivision of Madagascar, were randomly selected in a list of the *fokontany* surrounding the SHC: one close (i.e., centroid within a 2-km radius from the SHC) and the other further (i.e., centroid within a 10-15-km radius from the SHC), so as to include rural areas in the sample. In a given *fokontany*, investigators recorded the approximate number of households in each settlement and they included a number of households in each settlement proportionate to the total number of households in the whole *fokontany*. Households within a given settlement were selected according to a random pathway: a random point on a map of the settlement indicated the first household, and investigators then visited the second household on the right when stepping out from the previous household. Study teams were told to include at least 225 people of all age categories belonging to a minimum of 50 households in each of the 62 study sites, to make a total sample size of 13,950 individuals. If the study team could not include the expected number of individuals or households, or if they faced security and/or accessibility troubles, they investigated the closest *fokontany* in the list of randomized *fokontany*. Four teams of six surveyors and one supervisor investigated approximately eight study zones each, from September 2012 to January 2013. Coastal areas were investigated first, at the end of the dry season, in order to ensure accessibility to the study zones and to lessen the effect of seasonal transmission in the other transmission patterns. The latter were investigated at the beginning of the rainy season, i.e., just before or at the very beginning of the malaria transmission season.

#### Sample size calculation

The primary endpoint for sample size calculation was the proportion of the population infected by any *Plasmodium* species as diagnosed by RDT, and the proportion exposed to malaria control interventions. In a total sample size of 13,950, a two-sided 95% confidence interval for an expected proportion of population exposed to interventions of 50% and for an expected proportion of population infected by a *Plasmodium* of 5% will extend 0.8% and 0.4% from the observed proportion, respectively. In a sample size of at least 225 by site, a two-sided 95% confidence interval for an expected proportion of population exposed to interventions of 50% and for an expected proportion of population infected by a *Plasmodium* of 5% will extend 6.5% and 2.3% from the observed proportion in the sites, respectively.

#### Inclusion and data collection

Criteria for inclusion of individuals in the CSS were: i) member of or visitor in a household selected at random for inclusion in the CSS and whose head of household or his representative signed the household informed consent; ii) age ≥ six months; iii) signed individual informed consent including agreement for blood sampling; and, iv) ability to take a *per os* treatment in case of positive RDT. Geographic coordinates of each household were obtained using global positioning system (GPS).

The head of household or his representative answered a questionnaire about age and sex of all members of households and people having slept in the household the previous night (referred as ‘visitors’), housing features, living conditions, asset ownership, access to i) drugs retailer, ii) community health worker (CHW), iii) trained health staff, details of last IRS, bed net ownership, characteristics of the bed nets and use previous night, and verbal autopsy (VA) of household members deceased the previous year. Some characteristics of the bed nets (brand, positioning above sleeping places, and physical integrity) were checked visually by investigators. In one out of four households, the interviewee was asked to answer an additional questionnaire about social data: religion, ethnicity, social network, socio-economic self-perception, income, expenses, level of isolation, and migration. All included individuals were asked to answer a questionnaire about sociodemographic features, any fever episode during previous three months and case management in case of fever, bed net use over previous three months, anti-malarial drug intake over last year, mobility during previous three months, exposure to IEC messages about malaria (media, delay since last event), and, for females ≥15 years old, reproductive history, outcome (life/death) of all children, management of current or last pregnancy (antenatal care, IPTp).

In one out of four households, all individuals were asked about their professional activity and marital status, and an individual ≥15 years old was randomly selected to answer a knowledge, attitude and practice (KAP) questionnaire about health, pregnancy, fever, malaria, and malaria control interventions.

The VA questionnaire was designed from the World Health Organization’s international standard VA questionnaires [9], with added questions so as to collect all InterVA-3 indicators [10]. Criteria for answering the VA questionnaire were: specific informed consent, and delay since reported death between two and 12 months in order to respect a minimal mourning period while avoiding memory biases [11].

All answers were collected on notebooks with the help of a data entry form build on Microsoft Access® (Microsoft Office Professional Plus 2010, Redmond, WA, USA). Investigators were allowed to use paper questionnaires in case of software or hardware failure.

#### Collection of biological samples

All included individuals were punctured on finger or heel in order to perform a RDT (CareStart® Malaria, Access Bio Inc, Monmouth Junction, NJ, USA), to perform thick and thin blood smears, and to collect 100–500 μL capillary blood in an appropriate tube (MiniCollect® Z Serum Sep, Greiner Bio-One, Kremsmünster, Austria). Results of RDT were disclosed to individuals or their guardian after the interview, and people with a positive RDT result were proposed to take an anti-malarial treatment according to national guidelines. Thin smears were fixed with absolute methanol and serum tubes were centrifuged 10 min at 2,800 g on a daily basis. Serums tubes were kept at < 22°C until their transfer to the Institut Pasteur de Madagascar, where they were stored at -20°C.

#### Definition of variables

The individual coverage of LLINs was estimated by two means: i) whether the individual was listed among household members having slept under a LLIN the previous night according to the head of household or his representative, and, ii) according to the answer to the question “How many nights did you spend under a mosquito net during the last 3 months?” Answers to the latter question were categorized as following: “every night” versus any other answer (“1-6 time(s)/week”, “1-4 time(s)/month”, “less than once a month or never”). LLIN coverage was calculated for all individuals and not among individuals in household which owned a net only. In this manuscript the expression “coverage” refers to “use coverage” and not to “ownership coverage”.

A household was considered as covered by IRS if the head of household or his representative answered positively to the question “Did someone come in your household in the last 12 months to spray walls in order to fight against malaria?” and reported a delay since last IRS campaign ≤20 months so as to include only spraying during last campaign. For visitors, the IRS status at home was considered. Since the IRS campaign was late in the 2012–2013 rainy season, no household had been sprayed in the study sites at time of survey and no subset of households recently sprayed could be defined. In order to catch properly households covered by IRS, local designations (e.g., DDT) were used instead of, or in complement with, the term “spraying walls in order to fight against malaria”.

A woman was considered as covered by IPTp if she mentioned having taken at least two doses of sulphadoxine-pyrimethamine (SP) in the course of antenatal care during a ≥ four-month ongoing pregnancy or during her last pregnancy if she delivered within the previous 12 months. Males, females not of childbearing age (15–50 years old), nulliparous women, women pregnant < four months, and women who delivered more than 12 months previously were excluded from the calculation of IPTp coverage.

The following media were considered for the evaluation of exposure to IEC messages about malaria: radio (≤one month, >one to ≤ four months, or > four months since last message heard about malaria), poster (≤one month, >one to ≤ four months, or > four months since last poster seen), mobile video unit (MVU) (≤one year or > one year since last seen), television (≤four months or > four months since last message seen), leaflet or written press article (≤one year or > one year since last message read), and other media/exhibitions: whatever the delay spent since last event (e.g., *hiragasy* (traditional malagasy theatre), puppets, theatre, etc.).

A fever episode was defined as the self-report of any clinical sign of fever (e.g., hot body feeling, shivering) within the previous three months, whether or not accompanied by other clinical signs. A fever case management was defined as ‘appropriate’ as regards malaria if: i) either the patient underwent malaria diagnosis (RDT or microscopy, whether the result was negative or positive) by a trained health staff or a CHW (i.e*.*, he/she was supposed to be treated by ACT in case of diagnosis of *Plasmodium* infection), or ii) he/she took ACT (whether prescribed or self-administered) as presumptive treatment when he/she could not have access to malaria diagnosis. A recent uptake of anti-malarial drugs was defined as the treatment of last fever episode with anti-malarial drugs or self-report of uptake of anti-malarial drugs during three months before the interview.

Household socio-economic status (SES) was calculated using the first principal component score based on variables related to house size, living conditions (toilet, waste, water, lighting, cooking fuel), asset ownership, and household utilities. The scores were converted to wealth quintiles for analysis (Additional file 1). Housing construction materials (walls, roof, floor) and presence of holes in housing structure (i.e., lack of mosquito-proof housing conditions) were kept apart from the principal component analysis. The head of household or his representative was also interviewed about domestic use of other insecticides than IRS inside the household, i.e. aerosol sprays, coils, or agricultural insecticides.

#### Data processing and analysis

##### GIS data processing

A raster image of the density of population in Madagascar was obtained from WorldPop/AfriPop database [12]. WGS 84 image was converted to UTM 38S so as to reconstruct a 100*100 m grid by resampling closest pixel from WGS 84. Then, for each household and the centroid of each study site of the CSS, the population value was obtained. Cut-offs to classify the population density of a given location as high, intermediate or low were determined by comparison of population densities and landscape characterized by surveyors when conducting CSS (‘urban’, ‘town or suburban’ or ‘rural’). All pixel values corresponding to the territory of Madagascar were counted in order to determine the proportion of the population in the country living in each of these three categories of population density.

##### Weights and sample design

In order to estimate standardized proportions representative of the situation in the whole country, counts and proportions and their confidence intervals were weighted to account for the complex sample design and for the adjustment on i) gender and age categories, according to data collected during the previous Demographic and Health Survey in 2008–2009 [13]; ii) malaria transmission pattern according to population data provided by the UN Office for the Coordination of Humanitarian Affairs [14]; and, iii) population density categories as described above, using the *survey* package in R version 3.0.2 [15]. Significance of the differences between proportions was calculated using χ^2^ test as it applies to both crude and weighted proportions, but using Rao and Scott corrections in the latter situation [15], and considering a 5% significance threshold.

##### VA data processing

Although the VA questionnaire was based on InterVA-3, VA data were processed with the InterVA-4 software, given the improvements made since the previous version [16]. This software addresses up to three most probable causes – or an indeterminate diagnostic – to each death in the database and the likelihood associated with each cause. Deaths having multiple causes were attributed as fractional deaths for particular causes, proportional to cause-associated likelihoods, as suggested [16].

### Clinical malaria cases study

This clinical case study (CCS) aimed at evaluating the population affected by non-complicated malaria episodes. The design consisted of recruiting non-complicated malaria cases in health facilities belonging to the SHC network. As in the CSS, the sample of the CCS is made of clusters, stratified by transmission patterns.

#### Sample size calculation

The primary endpoint for sample size calculation was the exposure to malaria control interventions among patients presenting with clinical malaria due to any *Plasmodium* species. In a total sample size of 800, a two-sided 95% confidence interval for the proportion of population exposed to interventions will extend 3% from the observed proportion for an expected proportion of 50%.

#### Inclusion and data collection

Each of the 31 study zones was centred on one SHC. All these 31 SHCs were proposed to participate in the CCS. In the participating SHCs, all patients presenting with clinical malaria cases or their tutors submitted a short one-page questionnaire about socio-demographic data and exposure to malaria control interventions: LLIN, IRS and IPTp. Inclusion criteria were i) fever, i.e., axillary temperature ≥37.5°C [17] or self-reported symptoms of fever; ii) RDT or microscopy positive for any *Plasmodium* species; and, iii) informed consent of the patient or his/her tutor. Cases were retained in the database if they came from the same commune as individuals included in the CSS. Data were collected from September 2012 to August 2013.

#### Definition of variables

Exposure to malaria control interventions was defined the same way as for coverage in the CSS. The only exception was that a woman was exposed to IPTp if she mentioned having taken at least two doses of SP in the course of antenatal care during a ≥ four-month ongoing pregnancy or during last pregnancy if she delivered within the previous month (instead of “last 12 months”). For the comparison between cases and controls, the same definition was used in the CSS sample.

### Ethical considerations

Both CSS and CCS followed ethical principles according to the Helsinki Declaration. Informed consent was obtained from the individuals or the parents/tutors of the children before inclusion. The CSS and CCS were approved by the National Ethic Committee of the Ministry of Public Health of Madagascar (approval #CNE 57/MSANP/CE of 24 July, 2012).

## Results

### Cross-sectional survey

#### Population sampled

Among the 62 *fokontany* selected at random, 49 could be directly investigated; among the 13 other *fokontany*, three were rejected for security reasons, five could not be accessed by survey teams, e.g., if a river was flooding, in two cases the authorities refused to cooperate, e.g., because of their beliefs towards blood sampling, in other two cases a misunderstanding caused the team to investigate the wrong *fokontany,* and the last one was actually located in another district than the one being investigated. All 13 *fokontany* were replaced by other randomly selected *fokontany*. In the 62 study sites, the population of three hamlets refused to be investigated: one because of an ongoing event (funeral), another because of local taboos (*fady*) and the last one refused collection of blood without further explanation. In these cases, only the rest of the *fokontany* was investigated.

**Figure 2:**
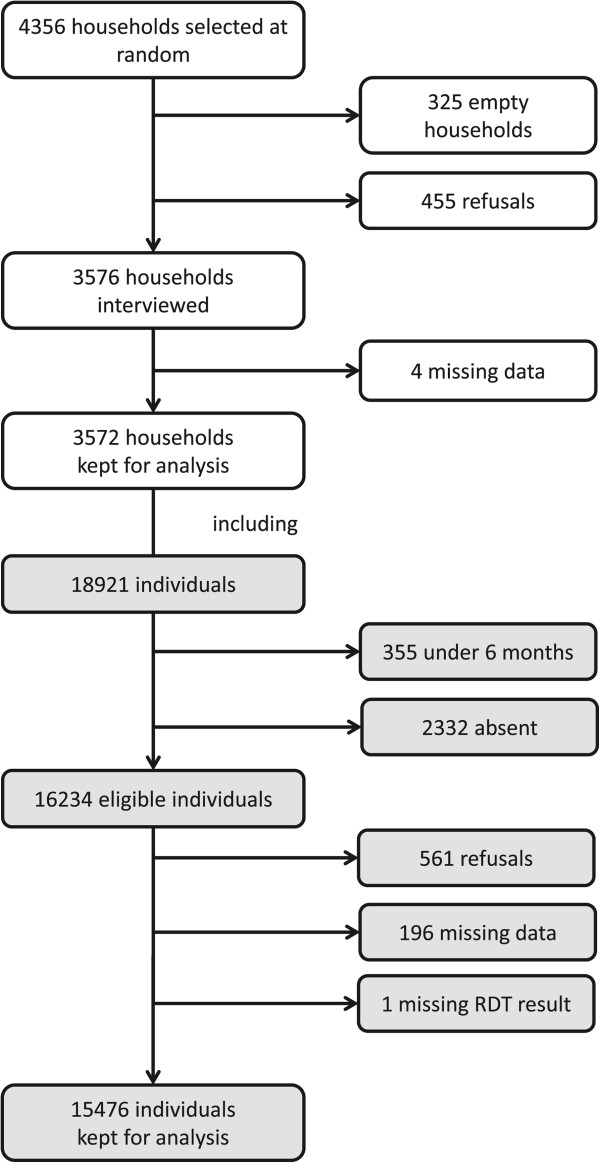
**Flow diagram of cross-sectional survey.**

Overall, 4,356 households were selected at random for interview. In 325 (7.5%), the household remained vacant during the survey. In 455 of the 4,031 remaining (11.3%), the head of household or his representative refused to participate (Figure 2). In 388 (85.3%) of the refusals, a reason was reported; most cited reasons for refusal were: not having time (35.3%), blood sampling refusal without further explanations (24.5%), blood sampling refusal for religious or taboos (*fady*) reasons (16.8%), and feeling healthy (i.e., they “don’t need medical investigation” 16.5%). All households that declined to participate were replaced with other households randomly selected from the same hamlet.

A total population of 19,921 individuals were members or visitors of the 3,572 households kept for analysis. Of the 16,234 eligible individuals, 561 (3.5%) either refused to be interviewed or sampled; in both cases, only sociodemographic data were collected. The final sample was 15,746 individuals.

Crude male/female ratio was 0.76; weighted male/female ratio was 0.98. Males were mainly under-represented in the crude sample in 20–39 year olds (Additional file 2). Crude proportion of visitors was 2.6% and weighted proportion was 2.5%. Regarding transmission patterns, the west was the most oversampled since 43.0% of the sample was located in this pattern while weighted proportion was 21.0%; Central Highlands and the East were the most subsampled since crude proportion were 12.8% and 22.3%, respectively, while weighted proportions were 32.4 and 28.1%. In the Fringe and the Southern patterns, crude proportions were 15.7 and 6.2% respectively, and weighted proportions were 13.1 and 5.4%.

Cut-offs of population density were set at two and 140 habitants/10,000 sq m. Considering this, 62.9% of the sample was estimated to live in rural areas (≤two habitants/10,000 sq m), 29.6% in small towns and suburban areas (>two and ≤140 habitants/10,000 sq m), and 7.6% in urban areas (>140 habitants/10,000 sq m). Weighted figures for these proportions were 59.1, 26.6 and 14.3%, respectively.

Low SES households were over-represented in the sample since the weighted proportions are inferior to the crude proportions in the two first quintiles, and the opposite is observed in the two last quintiles (Additional file 3). In the Central Highlands, high SES quintiles are prominent while the opposite is observed in the West, where more than half of the population belongs to the first (lowest) SES quintile. Wealthiest SES quintile is more frequently observed in settings where population density is intermediate or high (weighted proportions: 32.6 and 61.2%, respectively) than in low population density areas (weighted proportion: 9.6%,Additional file 4). Conversely the poorest quintile is almost absent from high-density areas (weighted proportion: 0.7%) while it represents 6.5 and 25.0% in intermediate-density and low-density areas, respectively (Additional file 4).

The largest group in terms of level of education among people ≥15 years old had accomplished primary school only (weighted proportion: 40.3%) and this situation was found in all transmission patterns (range of weighted proportions: 37.8-49.3%) except in the West (weighted proportion: 23.1%, Additional file 5). In the Western transmission pattern, the proportion of individuals ≥15 years old without any formal education was prominent (weighted proportion: 58.1%) while in other patterns this category was minor (range of weighted proportions: 4.9-18.1%, Additional file 5). The proportion of people ≥15 years old that had either no education or primary grade only was more important in areas of low population density (weighted proportion: 63.3%) than in other areas (weighted proportions: 41.3 and 32.9% in intermediate and high population density respectively, Additional file 4).

Among the 15,476 persons tested, 552 had a positive RDT result. Among these 552 samples, six slides were damaged and unreadable. Among the 546 remaining, all were examined by microscopy: 228 were found positive for *Plasmodium falciparum*, one positive for *Plasmodium vivax* and 317 negative. A sample of 459 slides from people with a negative RDT result was examined by microscopy: two were positive for *P. falciparum.* Overall, crude proportion of positive RDTs – or parasite rate (PR) – was 3.6% (95% CI [3.3-3.9]) and weighted PR was 3.1% (95% CI [2.1-4.5]). Weighted PR_2–10_ or PR between two and ten years old, an indicator for the intensity of transmission [18], was 4.2% (95% CI [2.7-6.6]). Another key indicator [19] is the proportion of children aged six to 59 months with malaria infection: the weighted value of this proportion was 2.8% (95% CI [1.8-4.4]) in the present study. The PR increased with age up to the age of nine then decreased progressively (p < 0.0001, Table 1). Infections in age groups between five and 19 years accounted for 57.8% of all *Plasmodium* infections. The weighted PR was higher in males (3.6%, 95% CI [2.3-4.9]) than in females (2.6%, 95% CI [1.5-3.7], p = 0.0004, Table 1). This difference in gender was observed in all age categories, except in under two year olds (Additional file 6).

**Table 1:** **Weighted proportions of individuals with a positive rapid diagnostic test by sociodemographic characteristics**

Variable	Category	N	n RDT positive	% [95% CI]
Age group	0-1 year	939	26	2.8 [1.0-4.6]
	2-4 years	1,480	41	2.8 [1.4-4.2]
	5-9 years	2,395	116	4.8 [2.5-7.2]
	10-15 years	2,233	105	4.7 [2.4-6.9]
	15-19 years	1,484	56	3.8 [2.0-5.5]
	20-39 years	4,016	94	2.4 [1.5-3.2]
	≥40 years	2,929	41	1.4 [0.7-2.0]
Sex	Male	7,644	275	3.6 [2.3-4.9]
	Female	7,832	204	2.6 [1.5-3.7]
SES quintiles	1st (poorest)	2,570	151	5.9 [3.2-8.6]
	2nd	2,658	114	4.3 [2.1-6.4]
	3rd	3,343	73	2.2 [1.1-3.3]
	4th	3,332	88	2.6 [1.5-3.7]
	5th (wealthiest)	3,574	54	1.5 [0.7-2.3]
Level of education (≥15 years old)	Unknown	22	0	0.0 [0.0-0.0]
	None	1,033	31	3.0 [1.5-4.4]
	Primary	3,395	85	2.5 [1.5-3.5]
	Lower secondary	2,492	51	2.0 [1.2-2.9]
	Upper secondary	1,093	21	1.9 [0.4-3.5]
	Tertiary	394	4	0.9 [0.0-2.2]
Transmission pattern	East	4,345	212	4.9 [2.0-7.8]
	West	3,255	151	4.6 [2.3-6.9]
	South	834	26	3.1 [0.0-7.1]
	Fringe	2,026	16	0.8 [0.0-1.7]
	Highlands	5,016	75	1.5 [0.4-2.6]
Population density	Low	9,147	340	3.7 [2.0-5.4]
	Intermediate	4,122	84	2.0 [0.9-3.2]
	High	2,207	55	2.5 [0.0-5.7]

Weighted PR was highest in the Eastern (4.9%) and Western (4.6%) transmission patterns. In the Southern transmission pattern, PR was 3.1%; in the Central Highlands and Fringe transmission patterns, it was 1.5 and 0.8%, respectively. These differences in PR between transmission patterns were significant (p = 0.0119). PR were higher in poorer SES quintiles (p = 0.0001) and in lower education levels although not significantly in the latter case (p > 0.1, Table 1). In rural areas (i.e., low population density), PR were higher than in the other two categories but this difference was not significant (p = 0.10, Table 1).

**Figure 3:**
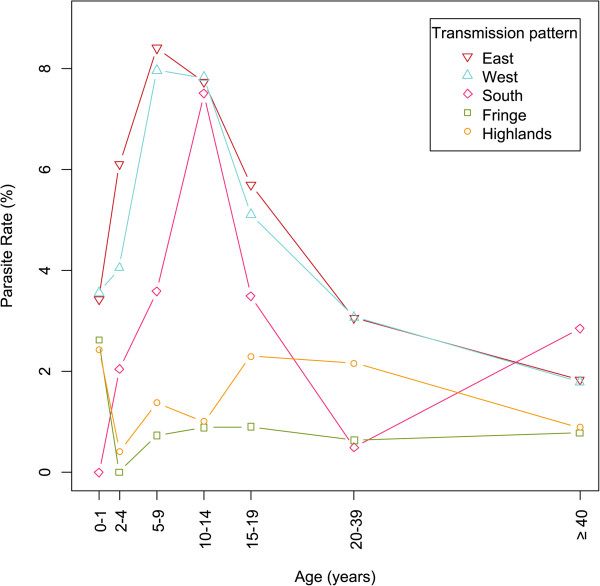
**Weighted parasite rates according to age and transmission patterns.** Weighted proportions of individuals with a positive RDT result by age categories centered on median age for the category, and by transmission pattern.

In the Western and Eastern transmission patterns, the PR was the highest among five to nine year olds (8.0 and 8.4%, respectively, Figure 3). In the South, PR was the highest among ten to 14 year olds (7.5%). In the Central Highlands and Fringe, PR was high among under one year olds (2.4 and 2.6%, respectively) but also higher among 15–19 year olds (2.3 and 0.9%, respectively) than among other age groups (Figure 3). There was a shift in higher PR toward the oldest in the lowest malaria transmission conditions.

### Coverage of malaria control interventions

#### Bed nets

**Figure 4:**
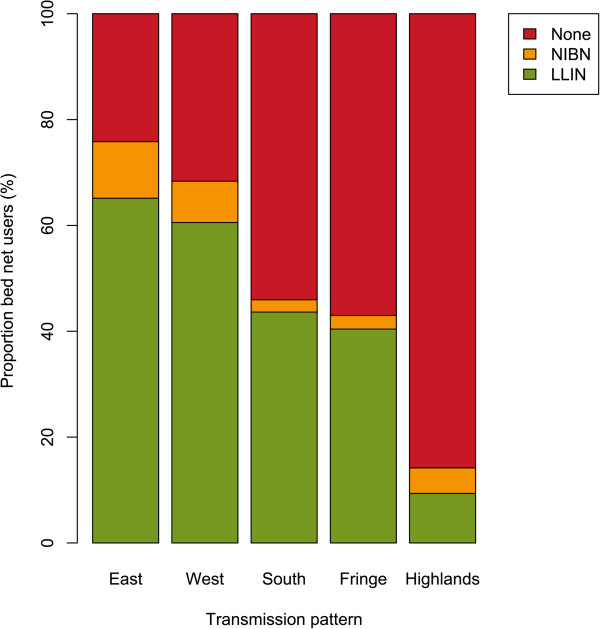
**Proportions of long-lasting insecticide net users, non-impregnated bed net users and no bed net users on the previous night.** Weighted proportions of individuals having used a LLIN, a NIBN or no bed net on the previous night, by transmission pattern.

The vast majority of bed nets were LLINs (5,461/6,267, 87.1%). Only nine bed nets were described as having been treated with insecticide at home. Most bed nets were used by one to three persons the previous night (65.8%) and a quarter (25.5%) remained unused. An important proportion of bed nets had been received from mass distribution (65.2% of all bed nets, 74.7% of LLIN). Figures of coverage according to transmission pattern or population density were similar whether measured as having slept under LLIN or non-impregnated bed net (NIBN) the previous night, or every night during the previous three months, although the proportion of every-night LLIN users was consistently lower than previous-night LLIN users (Additional file 7). In the Central Highlands, where no mass distribution of LLIN occurs, LLIN use was lower (9.4%, 95% CI [3.4-15.3]) than in other transmission patterns (57.2%, 95% CI [52.0-62.4], p < 0.0001, Table 2 and Figure 4).

**Table 2:** **Weighted proportions of long-lasting insecticidal net users on the previous night by sociodemographic characteristics**

Variable	Category	N	n LLIN users	% [95% CI]
Age group	0-1 year	939	494	52.6 [42.9-62.3]
	2-4 years	1,480	694	46.9 [37.1-56.7]
	5-9 years	2,395	965	40.3 [31.0-49.6]
	10-15 years	2,233	799	35.8 [26.9-44.6]
	15-19 years	1,484	475	32.0 [24.0-40.1]
	20-39 years	4,016	1,750	43.6 [34.4-52.7]
	≥40 years	2,929	1,276	43.6 [33.2-53.9]
Sex	Male	7,644	3,091	40.4 [31.7-49.2]
	Female	7,832	3,362	42.9 [33.7-52.2]
SES quintiles	1st (poorest)	2,570	1,272	49.5 [37.7-61.3]
	2nd	2,658	1,225	46.1 [34.0-58.2]
	3rd	3,343	1,222	36.5 [22.5-50.6]
	4th	3,332	1,321	39.6 [29.5-49.8]
	5th (wealthiest)	3,574	1,413	39.5 [28.0-51.1]
Level of education (≥15 years old)	Unknown	22	12	53.4 [25.4-81.3]
	None	1,033	546	52.9 [43.7-62.0]
	Primary	3,395	1,380	40.7 [29.6-51.7]
	Lower secondary	2,492	1,001	40.2 [30.8-49.6]
	Upper secondary	1,093	445	40.7 [30.8-50.6]
	Tertiary	394	117	29.6 [17.3-42.0]
Transmission pattern	East	4,345	2,830	65.1 [57.4-72.9]
	West	3,255	1,971	60.5 [54.6-66.4]
	South	834	364	43.6 [21.9-65.3]
	Fringe	2,026	819	40.4 [31.1-49.8]
	Highlands	5,016	470	9.4 [3.4-15.3]
Population density	Low	9,147	4,070	44.5 [33.6-55.4]
	Intermediate	4,122	1,608	39.0 [26.2-51.9]
	High	2,207	775	35.1 [16.2-54.0]

Crude proportion of LLIN users the previous night was 51.3% (95% CI [50.5-52.1]) and weighted proportion was 41.7% (95% CI [33.2-50.7]). LLIN use was highest among under-five age groups, and among adults over 19 years old (p < 0.0001, Table 2). Males used LLIN less frequently (40.4%) than females (42.9%, p = 0.007, Table 2). Proportions of LLIN users were higher in low SES categories and in low education levels, but these differences were significant only in the latter situation (p > 0.1 and p = 0.04, respectively, Table 2). Differences of coverage between categories of population density were not significant (p > 0.1, Table 2).

Key indicators of LLIN coverage [19] included: i) LLIN ownership, i.e. the proportion of households with at least one LLIN: 64% (weighted, 95% CI [52.9-73.8]); ii) the proportion of households with at least one LLIN for every two people: 22.7% (weighted, 95% CI [17.7-28.8]); iii) the proportion of population with access to a LLIN within their household, i.e., the proportion of individuals who could sleep under an LLIN if each LLIN in the household is used by two people [19]: 42.7% (weighted); iv) the proportion of children under five years old who slept under a LLIN the previous night: 49.1% (weighted, 95% CI [39.9-58.4 ]); v) the proportion of pregnant women who slept under an insecticide-treated net the previous night: 38.4% (weighted, 95% CI [29.0-48.8]); and, vi) the proportion of existing LLIN used the previous night: 64.9% (crude, 95% CI [63.7-66.1]).

The proportion of NIBN users was largely inferior to the proportion of LLIN users: crude proportion was 6.9% (95% CI [6.6-7.4]) and weighted proportion was 6.7% (95% CI [4.9-9.0]). The differences in age groups and sex were the same as with LLIN users, but proportions of NIBN users increase with SES quintiles, level of education and population density, contrary to what was observed with LLIN users (Additional file 8). Proportions of NIBN users differed also between transmission patterns, but the figure is different from proportions of LLIN users (Additional file 8 and Figure 4).

### IRS

The crude proportion of persons covered by IRS the previous 12 months was 27.6% (95% CI [26.9-28.3]) and weighted proportion was 30.2% (95% CI [21.4-40.8]). When considering only the zones targeted by IRS campaigns, i.e., Fringe, Southern and parts of Highlands and Western transmission patterns, the weighted proportion is 66.8% (95% CI [61.9-71.7]). Since all people living in a household having received IRS were considered as covered, there was no gradient between age groups. Differences between infants and older children or adults were significant (p = 0.01). There was no significant difference between sex (p > 0.1, Table 3). Urban areas, wealthiest SES quintiles and high levels of education had lower coverage proportions (p = 0.04, p = 0.003, and p = 0.02, respectively, Table 3).

**Table 3:** **Weighted proportions of sample covered by indoor residual spraying in the previous 12 months by sociodemographic characteristics**

Variable	Category	N	n IRS	% [95% CI]
Age group	0-1 year	939	250	26.6 [17.4-35.9]
	2-4 years	1,480	492	33.2 [23.2-43.2]
	5-9 years	2,395	770	32.2 [22.4-42.0]
	10-15 years	2,233	740	33.2 [22.2-44.1]
	15-19 years	1,484	448	30.2 [20.1-40.4]
	20-39 years	4,016	1,072	26.7 [16.7-36.6]
	≥40 years	2,929	904	30.9 [20.2-41.6]
Sex	Male	7,644	2,339	30.6 [20.4-40.8]
	Female	7,832	2,338	29.9 [20.2-39.5]
SES quintiles	1st (poorest)	2,570	874	34.0 [21.0-47.1]
	2nd	2,658	994	37.4 [24.3-50.5]
	3rd	3,343	1,364	40.8 [27.6-54.0]
	4th	3,332	1,056	31.7 [19.3-44.0]
	5th (wealthiest)	3,574	389	10.9 [1.9-19.8]
Level of education (≥15 years old)	Unknown	22	6	29.1 [8.2-49.9]
	None	1,033	304	29.4 [19.0-39.9]
	Primary	3,395	1,189	35.0 [23.3-46.8]
	Lower secondary	2,492	628	25.2 [14.9-35.5]
	Upper secondary	1,093	235	21.5 [9.6-33.5]
	Tertiary	394	61	15.5 [7.1-24.0]
Transmission pattern	East	4,345	166	3.8 [0.0-8.2]
	West	3,255	595	18.3 [5.6-31.0]
	South	834	566	67.8 [58.3-77.4]
	Fringe	2,026	1,186	58.6 [50.8-66.3]
	Highlands	5,016	2,163	43.1 [19.9-66.4]
Population density	Low	9,147	3,255	35.6 [23.8-47.4]
	Intermediate	4,122	1,173	28.5 [12.5-44.4]
	High	2,207	249	11.3 [4.0-18.5]

Key indicators of vector control coverage [19] also included: i) the proportion of households with at least one LLIN and/or sprayed by IRS in the previous 12 months: 78% (weighted, 95% CI [69.7-84.5]); and, ii) the proportion of households with at least one LLIN for every two people and/or sprayed by IRS within the previous 12 months: 47.5% (weighted, 95% CI [40.0-55.1]).

### Domestic use of insecticides

The crude proportion of persons living in households where they used other insecticides than IRS was 17.7% (95% CI [17.1-18.3]) and weighted proportion was 22.8% (95% CI [17.9-28.5]). Upper SES quintiles, high levels of education and central transmission patterns (Highlands, Fringe) displayed much higher proportions of domestic use of insecticides (p = 0.0003, p < 0.0001 and p < 0.0001, respectively, Table 4).

**Table 4:** **Weighted proportions of sample in households reporting domestic use of insecticides by sociodemographic characteristics**

Variable	Category	N	n	% [95% CI]
Age group	0-1 year	939	167	17.8 [12.5-23.1]
	2-4 years	1,480	284	19.2 [13.6-24.8]
	5-9 years	2,395	490	20.5 [14.9-26.0]
	10-15 years	2,233	557	24.9 [18.6-31.3]
	15-19 years	1,484	335	22.6 [17.1-28.0]
	20-39 years	4,016	968	24.1 [17.6-30.6]
	≥40 years	2,929	728	24.9 [19.0-30.7]
Sex	Male	7,644	1,858	24.3 [18.5-30.1]
	Female	7,832	1,671	21.3 [16.3-26.3]
SES quintiles	1st (poorest)	2,570	233	9.1 [3.6-14.5]
	2nd	2,658	429	16.1 [9.2-23.1]
	3rd	3,343	783	23.4 [14.4-32.4]
	4th	3,332	917	27.5 [18.9-36.1]
	5th (wealthiest)	3,574	1,168	32.7 [24.6-40.8]
Level of education (≥15 years old)	Unknown	22	1	2.9 [0.0-8.6]
	None	1,033	96	9.3 [5.8-12.7]
	Primary	3,395	706	20.8 [14.8-26.8]
	Lower secondary	2,492	672	27.0 [20.4-33.5]
	Upper secondary	1,093	382	34.9 [27.0-42.8]
	Tertiary	394	175	44.4 [28.2-60.7]
Transmission pattern	East	4,345	392	9.0 [4.4-13.7]
	West	3,255	445	13.7 [9.2-18.1]
	South	834	25	3.0 [1.3-4.8]
	Fringe	2,026	606	29.9 [22.7-37.1]
	Highlands	5,016	2,060	41.1 [35.8-46.3]
Population density	Low	9,147	1,878	20.5 [14.0-27.0]
	Intermediate	4,122	1,054	25.6 [15.7-35.4]
	High	2,207	597	27.0 [16.0-38.1]

Weighted numbers and proportions of individuals living in households that reported domestic use of other insecticides than IRS, according to sociodemographic characteristics.

### IPTp

The crude proportion of women having received ≥ two doses of IPTp among pregnant women over four months of gestational age or women of childbearing age (i.e., 15–49 years old) having delivered in previous 12 months was 23.0% (95% CI [20.3-26.0]), and weighted proportion was 21.5% (95% CI [17.2-26.5]). When considering only the zones targeted, i.e., all transmission patterns but Central Highlands, the weighted proportion was 25.3% (95% CI [20.0-30.5]); in the South, coverage was also lower than in other transmission patterns (p = 0.0005, Table 5). Age, SES, education, and population density were not significantly associated with IPTp coverage (p > 0.1, p > 0.1, p > 0.1, and p = 0.07, respectively, Table 5). The proportion of women having received ≥ three doses of IPTp among women of childbearing age having delivered in previous 24 months was 8.4% (weighted, 95% CI [6.7-10.6]).

**Table 5:** **Weighted proportions of women receiving two doses of intermittent preventative treatment in pregnancy by sociodemographic characteristics**

Variable	Category	N	n IPTp	% [95% CI]
Age group	15-24 years	305	62	20.2 [14.1-26.3]
	25-34 years	269	62	23.2 [16.9-29.5]
	35-49 years	111	23	20.8 [11.4-30.2]
SES quintiles	1st (poorest)	156	37	23.9 [16.7-31.1]
	2nd	130	31	23.9 [12.4-35.4]
	3rd	144	21	14.5 [8.4-20.7]
	4th	129	32	24.4 [16.5-32.4]
	5th (wealthiest)	126	26	20.9 [14.5-27.2]
Level of education	Unknown	0	0	-
	None	114	18	15.7 [8.3-23.1]
	Primary	307	71	23.2 [17.0-29.4]
	Lower secondary	196	39	19.9 [12.9-27.0]
	Upper secondary	51	17	33.6 [18.5-48.7]
	Tertiary	16	2	9.8 [0.0-25.5]
Transmission pattern	East	206	55	26.7 [16.9-36.4]
	West	147	35	23.5 [17.1-29.9]
	South	52	5	10.3 [7.0-13.5]
	Fringe	100	33	32.7 [23.9-41.5]
	Highlands	181	20	10.9 [5.8-16.0]
Population density	Low	418	104	24.8 [18.5-31.2]
	Intermediate	182	28	15.3 [10.1-20.6]
	High	85	15	18.0 [8.4-27.7]

Weighted numbers and proportions of women having received two doses of IPTp among pregnant women over four months of gestational age or women having delivered in previous 12 months, according to sociodemographic characteristics.

### IEC campaigns

Most cited medium for IEC messages about malaria was the radio (weighted proportion, 37.1%, 95% CI [32.5-41.8]), and second most cited was posters (19.6%, 95% CI [14.8-25.5]). Other media include television (13.4%, 95% CI [10.0-17.9]), information meetings (6.1%, 95% CI [4.8-7.6]), reading documents and/or articles (3.5%, 95% CI [2.6-4.7]), MVU (2.4%, 95% CI [1.6-3.5]), and other exhibitions such as *hiragasy*, i.e., traditional theatre (1.4%, 95% CI [0.8-2.2]). Exposure to radio IEC messages about malaria is significantly lower in younger ages, lower education levels and urban areas (p < 0.0001, p < 0.0001 and p = 0.004, respectively, Table 6); differences between sex and transmission patterns are not significant (p = 0.06 and p > 0.1, respectively, Table 6). Exposure to IEC messages was significantly higher in upper SES for most media (p = 0.01, p < 0.0001, p = 0.001, p = 0.007 for radio, TV, documents, and MVU respectively), non-significantly higher in upper SES for posters and ‘others’ (p = 0.1 and p = 0.05, respectively), and non-significantly higher in lower SES for information meetings (p = 0.07, Table 6). Exposure to information meetings was significantly higher in areas of low population density (p = 0.04, Table 6).

**Table 6:** **Weighted proportions of individuals having received information, education and communication messages about malaria by sociodemographic characteristics**

Variable	Category	Weighted N	Radio	Posters	Television	Information meetings	Documents/articles	MVU	Others (hiragasy,…)
Age category	15-19 years	1,484	25.8 [21.0-30.6]	15.9 [11.2-20.5]	10.0 [6.9-13.2]	2.7 [1.7-3.8]	4.6 [2.8-6.5]	3.2 [1.8-4.5]	0.7 [0.2-1.2]
	20-39 years	4,016	38.0 [33.5-42.6]	20.6 [14.9-26.3]	13.6 [9.7-17.6]	6.4 [4.7-8.1]	3.1 [1.9-4.2]	2.6 [1.5-3.8]	1.4 [0.6-2.2]
	≥40 years	2,929	41.4 [35.6-47.2]	20.1 [13.9-26.3]	14.8 [9.8-19.9]	7.3 [5.6-9.0]	3.5 [2.4-4.7]	1.7 [1.0-2.4]	1.6 [0.6-2.6]
Sex	Male	4,119	38.7 [33.3-44.1]	20.1 [14.8-25.4]	13.1 [9.2-17.0]	4.5 [3.3-5.8]	4.0 [2.7-5.4]	2.9 [1.8-4.0]	1.2 [0.6-1.8]
	Female	4,311	35.5 [30.9-40.1]	19.1 [13.4-24.8]	13.7 [9.5-18.0]	7.5 [5.8-9.3]	3.0 [2.1-3.9]	1.9 [0.9-2.8]	1.5 [0.7-2.3]
SES quintiles	1st (poorest)	1,179	24.7 [19.4-30.0]	12.3 [7.5-17.2]	1.0 [0.4-1.7]	7.5 [5.2-9.8]	0.5 [0.1-0.9]	1.2 [0.0-2.5]	0.4 [0.0-0.8]
	2nd	1,319	30.6 [25.4-35.7]	14.2 [10.3-18.1]	1.5 [0.8-2.2]	6.6 [4.3-8.9]	2.2 [1.0-3.3]	0.8 [0.1-1.5]	0.9 [0.0-1.8]
	3rd	1,817	40.0 [34.8-45.2]	21.1 [14.7-27.5]	4.2 [2.6-5.8]	7.2 [5.4-9.1]	3.5 [2.1-4.9]	1.6 [0.7-2.5]	1.4 [0.4-2.4]
	4th	1,857	42.5 [36.1-48.9]	23.9 [14.5-33.3]	13.9 [10.4-17.5]	6.0 [3.9-8.1]	4.3 [2.8-5.8]	3.9 [1.9-5.9]	2.5 [0.8-4.2]
	5th (wealthiest)	2,257	40.5 [29.2-51.8]	21.7 [11.0-32.4]	33.9 [31.0-36.8]	4.2 [2.5-5.8]	5.2 [2.7-7.7]	3.3 [1.5-5.1]	1.2 [0.1-2.2]
Level of education	Unknown	22	17.3 [0.0-36.6]	12.4 [0.0-31.0]	12.8 [0.0-31.6]	8.5 [0.0-18.6]	0.0 [0.0-0.0]	0.0 [0.0-0.0]	0.0 [0.0-0.0]
	None	1,033	22.6 [17.3-27.8]	5.8 [3.2-8.4]	2.2 [0.8-3.7]	5.6 [3.3-7.8]	0.0 [0.0-0.0]	0.4 [0.0-0.7]	0.5 [0.0-1.0]
	Primary	3,395	33.1 [29.4-36.7]	14.6 [11.0-18.3]	6.2 [3.9-8.4]	5.3 [4.1-6.5]	1.5 [0.8-2.1]	1.3 [0.6-2.0]	1.3 [0.6-2.1]
	Lower secondary	2,492	39.2 [32.9-45.5]	22.8 [15.5-30.1]	17.3 [13.1-21.4]	7.3 [4.8-9.9]	5.2 [3.7-6.6]	2.9 [1.4-4.4]	1.4 [0.4-2.5]
	Upper secondary	1,093	52.2 [43.9-60.5]	33.5 [21.5-45.5]	26.3 [21.9-30.6]	4.9 [2.9-6.9]	6.8 [4.2-9.5]	4.8 [2.6-6.9]	2.1 [0.7-3.4]
	Tertiary	394	54.9 [40.3-69.5]	39.9 [19.7-60.1]	45.6 [37.9-53.3]	9.2 [5.2-13.3]	10.8 [3.9-17.7]	6.8 [0.0-13.7]	1.4 [0.0-2.7]
Transmission pattern	East	2,396	36.7 [28.9-44.5]	16.9 [10.5-23.3]	12.2 [6.4-17.9]	6.5 [4.8-8.3]	3.0 [1.7-4.4]	3.2 [0.7-5.7]	1.5 [0.3-2.7]
	West	1,746	38.4 [34.3-42.6]	20.1 [15.8-24.4]	14.6 [8.9-20.4]	5.6 [4.3-7.0]	3.8 [2.4-5.2]	3.1 [1.8-4.4]	0.7 [0.3-1.1]
	South	332	11.7 [0.6-22.8]	4.5 [0.3-8.8]	1.7 [0.0-3.7]	13.0 [7.7-18.2]	1.1 [0.0-2.4]	1.0 [0.0-2.1]	0.2 [0.0-0.6]
	Fringe	1,076	41.1 [33.4-48.8]	20.1 [13.7-26.4]	8.6 [2.3-14.8]	8.8 [5.8-11.7]	2.9 [1.3-4.5]	2.1 [0.3-4.0]	1.9 [0.1-3.6]
	Highlands	2,880	38.0 [26.9-49.1]	23.1 [8.5-37.6]	16.9 [8.0-25.8]	4.2 [1.4-6.9]	4.2 [1.7-6.7]	1.5 [0.2-2.9]	1.6 [0.0-3.2]
Population density	Low	4,838	38.4 [33.1-43.7]	20.9 [15.2-26.7]	7.6 [3.3-11.9]	6.9 [5.4-8.5]	3.7 [2.3-5.2]	1.6 [0.8-2.4]	1.1 [0.5-1.7]
	Intermediate	2,223	41.1 [35.3-47.0]	23.7 [15.1-32.4]	18.1 [13.4-22.9]	6.1 [3.9-8.3]	3.5 [2.1-4.9]	3.9 [1.1-6.6]	2.3 [0.9-3.7]
	High	1,368	25.7 [18.9-32.5]	8.0 [4.2-11.8]	26.3 [23.5-29.2]	3.0 [1.0-4.9]	2.8 [0.9-4.7]	2.8 [1.6-3.9]	0.6 [0.0-1.6]

Total weighted numbers of individuals ≥15 years old and proportions having received IEC messages about malaria, per media and per sociodemographic characteristics.

### Fever case management

The weighted proportion of people who declared having presented a fever in previous three months was 12.1% (95% CI [10.5-14.0]). Data about 1,380 fever case managements was collected. Among these, 37.6% sought advice or treatment from a professional health worker or a CHW, 13.8% had a finger or heel stick for malaria diagnosis, and 17.6% received a treatment against malaria. Among people having consulted at a health centre or hospital, 71.5% resorted to the public sector. Among persons having received an anti-malarial, 31.4% received ACT. Overall, whether febrile or not, 2.7% of the individuals interviewed declared intake of a treatment against malaria in the previous three months. The weighted proportion of fever case managements considered as appropriate as regards malaria was 15.8% (95% CI [12.7-19.5]). The crude proportion was 17.5% (95% CI [15.6-19.7]). Proportions of appropriate case managements (ACM) were significantly higher in younger ages and in transmission patterns with higher PRs (p = 0.008 and p = 0.046, respectively, Table 7), but differences between sex, SES quintiles, level of education, and population density were not significant (all p > 0.1, Table 7).

**Table 7:** **Weighted proportions of appropriate case managements by sociodemographic characteristics**

Variable	Category	N fever	n ACM	% [95% CI]
Age group	0-1 year	169	35	20.8 [12.6-28.9]
	2-4 years	216	46	21.1 [14.0-28.2]
	5-9 years	193	37	19.1 [11.9-26.4]
	10-15 years	109	23	20.7 [12.1-29.3]
	15-19 years	82	8	9.3 [2.3-16.4]
	20-39 years	310	35	11.4 [7.5-15.2]
	≥40 years	274	31	11.2 [7.1-15.4]
Sex	Male	675	108	15.9 [12.7-19.2]
	Female	679	106	15.7 [11.2-20.1]
SES quintiles	1st (poorest)	201	33	16.3 [11.6-21.1]
	2nd	243	46	18.8 [12.0-25.6]
	3rd	291	50	17.3 [10.0-24.6]
	4th	303	40	13.3 [8.8-17.9]
	5th (wealthiest)	315	45	14.2 [9.8-18.5]
Level of education (≥15 years old)	Unknown	2	0	18.6 [0.0-52.1]
	None	88	9	10.5 [1.6-19.4]
	Primary	253	29	11.6 [7.2-15.9]
	Lower secondary	232	25	10.6 [4.8-16.5]
	Upper secondary	75	8	10.4 [2.0-18.7]
	Tertiary	16	2	14.1 [0.2-28.1]
Transmission pattern	East	473	92	19.4 [13.0-25.8]
	West	316	60	19.1 [14.7-23.5]
	South	66	14	20.4 [6.7-34.2]
	Fringe	138	17	12.0 [6.3-17.7]
	Highlands	361	32	8.8 [3.0-14.6]
Population density	Low	753	129	17.2 [11.8-22.5]
	Intermediate	343	45	13.0 [8.6-17.4]
	High	257	40	15.5 [11.8-19.1]

Weighted numbers and proportions of appropriate case managements (ACM) among persons who presented a fever last 3 months, according to socio-demographic characteristics.

Data about case management in 258 children under five years old who had fever in the previous two weeks were collected. Key indicators of case management [19] also included: i) the proportion of children under five years old who had fever in the previous two weeks and who had a finger or heel stick: 14.2% (weighted, 95% CI [8.3-23.1]); ii) the proportion of children under five years old who had fever in the previous two weeks for whom advice or treatment was sought from a professional health worker or a CHW: 38.8% (weighted, 95% CI [32.1-45.9]); and, iii) the proportion receiving ACT, among children under five years old who had fever in the previous two weeks who received any anti-malarial drugs: 37.2% (weighted, 95% CI [16.3-64.4]).

### Mortality attributable to malaria

**Figure 5:**
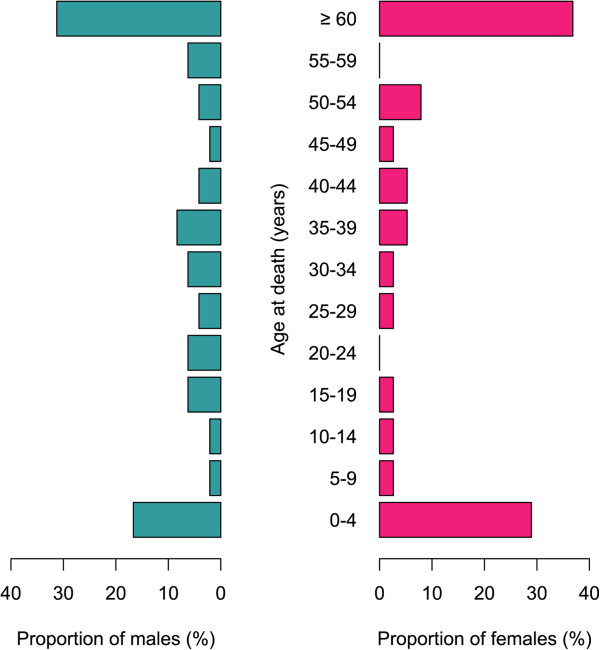
**Age and sex profile of 86 deaths investigated by verbal autopsy.**

Among the 3,516 households interviewed for the occurrence of death in the household during the previous 12 months, 114 deaths were noted. Since 18,597 individuals were included in these 3,516 household, crude mortality rate is evaluated to be 6.1% (95% CI [5.1-7.3]). No explicit refusal to conduct VA was noted, but in eight cases, data were missing. In 20 cases, the death occurred outside the two to 12 months’ delay before the date of interview and no VA was conducted. The VA data from 86 deaths was collected, most of them occurring in elders or in under-fives (Figure 5).

After analysis by InterVA-4 software, the cause-specific mortality fraction (CSMF) of malaria proportional to cause-associated likelihood was 14.0% (95% CI [7.7-23.5]). Malaria ranked second CSMF after cardiovascular diseases and was attributed as most probable cause of death in 12 cases and as second most probable cause of death in two cases (Table 8). Apart from infants and neonates, there were deaths attributable to malaria in all age groups, but CSMF were higher in the one to four and five to 14 years age groups (p < 0.0001, Table 8). CSMF was 43.6% (95% CI [22.3-67.4]) before the age of five, and 8.7% (95% CI [3.5-18.8]) after that age. Only half of the malaria-attributed deaths occurred before the age of five (7/14).

**Table 8:** **Age distribution of deaths and deaths attributable to malaria**

Age at death	Total N	Attributable to malaria		
		N 1 ^st^ cause	N 2 ^nd^ cause	CSMF (% [95% CI])
<1 year	8	0	0	0.0 [0.0-40.2]
1-4 years	11	6	1	61.7 [30.1-86.5]
5-14 years	4	2	0	48.8 [13.8-85.0]
15-60 years	34	3	1	8.7 [2.2-24.6]
≥60 years	29	1	0	2.6 [0.1-18.5]
Total	86	12	2	14.0 [7.7-23.5]

Total number of deaths, number of deaths for which malaria was attributed as first and second cause of death by InterVA-4 software, and CSMF proportional to likelihood, per age group and overall.

### Clinical case study

**Figure 6:**
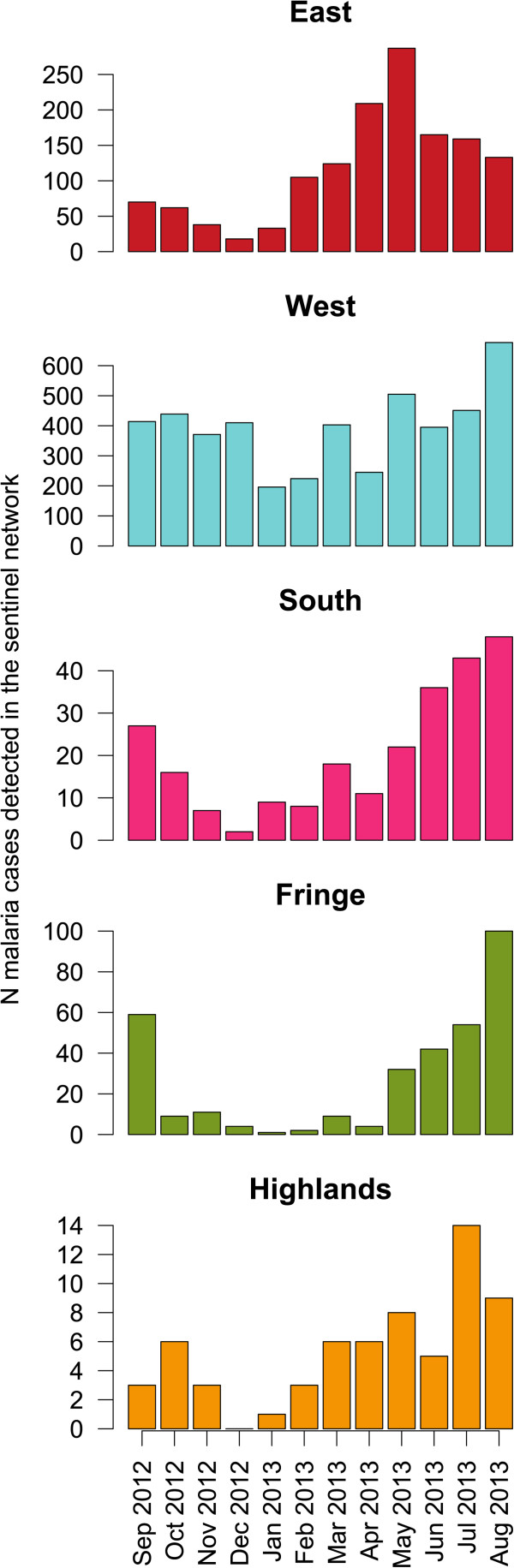
**Monthly number of malaria cases detected in the fever sentinel network.** Monthly number of malaria cases detected in SHCs during the period of data collection, per transmission pattern.

From September 2012 to August 2013, 6,771 malaria cases were observed in all 34 SHCs (Figure 6). Among those cases, 4,223 individual sheets (62.4%) were filled. Twenty-eight SHCs participated in the CCS and sent 1,585 filled questionnaires, among which 923 (58.2%) were individuals coming from same communes as individuals included in the CSS. Among those, 818 questionnaires (88.6%) were correctly filled, from 25 SHCs.

In transmission patterns with high incidence, the age of clinical cases was younger than in transmission patterns with lower incidence (p < 0.0001, Table 9). People above the age of five accounted for 79.7% of all clinical cases. Males were more affected than females (p = 0.01), and there were no significant differences between transmission patterns (p > 0.1, Table 9). The proportion of LLIN users on the previous night was 52.1% (95% CI [48.6-55.5]), and the difference between transmission patterns was close to significance threshold (p = 0.0503, Table 9). Excluding Central Highlands, where no LLIN distributions occurred, proportion of LLIN users among clinical cases was 52.4% (95% CI [48.9-55.9]). The overall proportion of clinical cases living in households having received IRS the previous year was 11.6% (95% CI [9.4-13.9]), and this proportion in IRS-targeted zones was 48.4% (95% CI [41.0-55.8]). There were 11 women pregnant (second or third trimester) or having delivered the previous month; three of whom had received IPTp (Table 9).

**Table 9:** **Distribution of sentinel health centres and clinical malaria cases per transmission patterns**

		Transmission pattern					
Variable	Category	East	West	South	Fringe	Highlands	Total
No. SHC		8	11	1	2	3	25
No. clinical cases		249	497	8	57	7	818
Age category	0-1 year	25 (10.0)	22 (4.4)	0 (0.0)	9 (15.8)	0 (0.0)	56 (6.8)
	2-4 years	34 (13.7)	63 (12.7)	1 (12.5)	12 (21.1)	0 (0.0)	110 (13.4)
	5-9 years	51 (20.5)	104 (20.9)	1 (12.5)	15 (26.3)	0 (0.0)	171 (20.9)
	10-15 years	56 (22.5)	105 (21.1)	3 (37.5)	4 (7.0)	0 (0.0)	168 (20.5)
	15-19 years	38 (15.3)	75 (15.1)	2 (25.0)	4 (7.0)	1 (14.3)	120 (14.7)
	20-39 years	26 (10.4)	96 (19.3)	1 (12.5)	11 (19.3)	6 (85.7)	140 (17.1)
	≥40 years	19 (7.6)	32 (6.4)	0 (0.0)	2 (3.5)	0 (0.0)	53 (6.5)
Sex	Male	132 (53.0)	276 (55.5)	6 (75.0)	28 (49.1)	4 (57.1)	446 (54.5)
	Female	117 (47.0)	221 (44.5)	2 (25.0)	29 (50.9)	3 (42.9)	372 (45.5)
LLIN use the previous night	Yes	140 (56.2)	250 (50.3)	2 (25.0)	33 (57.9)	1 (14.3)	426 (52.1)
	No	109 (43.8)	247 (49.7)	6 (75.0)	24 (42.1)	6 (85.7)	392 (47.9)
IRS the previous year	Yes	3 (1.2)	68 (13.7)	5 (62.5)	18 (31.6)	0 (0.0)	94 (11.6)
	No	244 (98.8)	427 (86.3)	3 (37.5)	39 (68.4)	6 (100.0)	719 (88.4)
Received IPTp	Yes	2 (40.0)	1 (20.0)	0	0 (0.0)	0	3 (27.3)
	No	3 (60.0)	4 (80.0)	0	1 (100.0)	0	8 (72.7)

Number of participating SHC, total number of clinical cases analysed, and number and proportion of clinical cases according to transmission patterns, and totals.

## Discussion

These results mainly confirm figures found in Malaria Indicator Survey (MIS) in Madagascar or other surveys, e.g., PRs are higher in Eastern and Western transmission patterns, PR increases with age up to the age of five, and coverage of malaria control interventions remain suboptimal, especially IPTp [7, 20]. These results also confirm that SES, education and environmental factors, such as population density, are all related to malaria infection and are closely intertwined [21]. Despite better LLIN coverage of individuals belonging to lower SES or educational level, social inequalities regarding malaria infection are striking. These inequalities can be explained by better access to prophylaxis (e.g., purchase and use of NIBN, as results show) and treatment, better housing quality and better nutritional status [21], and deserve specific investigation. It cannot be excluded that the slightly higher rates of appropriate fever case management in poorer SES quintiles are due to indication bias since higher ACM rates are observed in high-transmission areas, where higher proportions of low SES quintiles were observed. The lower IRS coverage in high-density areas is related to the absence of IRS campaigns in the centres of main cities in the Central Highlands.

PR were inferior to figures observed during MIS in 2011 and 2013 (Table 10). Two main factors could explain this difference: i) in the present study, the sampling took place between September and January, i.e., at the end of the dry season and beginning of rainy season, while MIS took place in November-December [7] or February-March [20], i.e., during the rainy season when incidence of malaria is higher; ii) the present study covers the whole country, including high altitude areas and big city centres that are supposed to be malaria free and not investigated in MIS [7, 20]. Seasons and inclusion of all areas also explains the lower LLIN coverage that was observed (Table 10), since LLIN use may depend on density of mosquitoes according to qualitative results [Mattern C, Raboanary E, Kesteman T, Rogier C, Piola P, Pourette D: Malaria is not a Problem. Consequences on Fever Case Management and Use of Bed Nets in Madagascar. Manuscript submitted] and drops in the Central Highlands where there is no distribution of LLIN. This result also mitigates the optimism arising from MIS results as regards LLIN coverage since a seasonal decrease in LLIN use can contribute to *Plasmodium* transmission during the dry season in areas where *Anopheles* breeding sites could persist, e.g., in rice fields and permanent water collections. These results regarding IRS, IPTp and fever case management stand between or are close to MIS results (Table 10).

**Table 10:** **Main indicators found from the Malaria Indicator Survey and the present study**

Indicator	MIS 2011	MEDALI 2012	MIS 2013
% RDT positive (PR) among under 5	8.7	3.1	10.0
% LLIN users the previous night - overall	68.3	41.7	54.5
% LLIN users the previous night - target zones	82.4	57.2	76.8
% covered by IRS previous 12 months - overall	43.1	30.2	29.8
% covered by IRS previous 12 months - target zones	81.5	66.8	59.7
% pregnant women covered by IPTp - overall	19.5	21.5	18.4
% pregnant women covered by IPTp - target zones	22.0	25.3	20.9
% under-fives with fever who had finger/heel stick	6.2	14.2	13.4
% ACT among anti-malarial treatments in under-fives	19.5*	37.2	54.0*

Main indicators found from MIS 2011, the present study (MEDALI, 2012) and MIS 2013 in Madagascar [7, 20]. *calculated from data disclosed in reports.

At first glance, this study may look like a MIS. Nevertheless, this study presents noteworthy differences. First, the population as a whole was investigated, not excluding geographical regions as it is done in MIS in Madagascar [7, 20], and above all not focusing on under-fives and women. Therefore it appeared that individuals between five and 19 years old presented higher PR, and represent probably a major reservoir and should be the main target of malaria control interventions in the context of pre-elimination in Madagascar. Concurrently, this age group presented lower coverage of LLIN, indicating a way to reduce this reservoir if coverage with LLIN was uniform between age groups. This result confirms that control programmes and evaluation surveys such as MIS should drop target groups (e.g. under-fives and pregnant women) and encompass the whole population when it comes to reducing transmission. While this policy is stated in international guidelines [22], national or local policies may stick to target groups [1]. In areas other than Madagascar, characterized by higher transmission, the reservoir may involve younger age groups [23]. It was evidenced that males of nearly all age groups were more frequently infected by *Plasmodium* as measured by RDT. This phenomenon could be explained by a biological susceptibility to infection, e.g., since lower serum iron concentration in females might protect them from *Plasmodium* infection [24], but also by differences in behaviour between gender that could deserve special attention as a target for IEC messages. Big city centres in Central Highlands and areas located at high altitude were included for two main reasons: i) SHCs in these areas detect few, but some, malaria cases that are not imported [personal communication, Randrianasolo L, Piola P, and Boyer S, Institut Pasteur de Madagascar]; and, ii) severe outbreaks occurred in these areas in the late 1980s and may occur again [25]. Recent detection of locally acquired clinical cases of malaria in the surroundings of the capital Antananarivo during the dry season of 2014 [personal communication, Randrianasolo L, Piola P, and Boyer S, Institut Pasteur de Madagascar] confirms that *a priori* any region of the country is permanently malaria-free.

Another major difference from MIS is the collection of data about malaria morbidity and mortality. As far as we know, this study is the first to encompass investigation of *Plasmodium* infection, control interventions, clinical cases, and malaria-attributable mortality. Results show that, similar to age profiles of PR, clinical cases occur in under-fives in 20.3% of cases, and that half the casualties could involve people over the age of five. Data from the CCS showed that in target zones coverage of LLIN and IRS were lower in clinical cases (52.4 and 48.4%, respectively) than in the overall population (57.2 and 66.8%, respectively). Adjusted ORs and protective effectiveness of malaria control interventions will be provided in a separate publication.

Results from a concurrent qualitative survey in four of the 31 settings investigated in the present survey suggest that the effect of IEC campaigns might be mitigated by predominance of informal channels in what people retain about malaria [Mattern C, Raboanary E, Kesteman T, Rogier C, Piola P, Pourette D: Malaria is not a Problem. Consequences on Fever Case Management and Use of Bed Nets in Madagascar. Manuscript submitted], which is consistent with the apparent low coverage of conventional media that was observed in quantitative results, and with findings in other countries [26]. The same qualitative survey also suggest that mosquitoes density affect LLIN use [Mattern C, Raboanary E, Kesteman T, Rogier C, Piola P, Pourette D: Malaria is not a Problem. Consequences on Fever Case Management and Use of Bed Nets in Madagascar. Manuscript submitted], which can explain that LLIN use in MIS is higher than in the present study (Table 10), since the presence of mosquitoes varies with seasons.

In Madagascar, hospital mortality attributable to malaria ranked fourth in 2011 [27]; in the present study it ranked second in overall mortality. In 2011, hospital mortality attributable to malaria was 8% among under-fives and 2% above this age; in the present study, overall mortality attributable to malaria in these age groups were 43.6 and 8.7%, respectively. These differences are, at least partially, explained by the fact that most malaria-attributable deaths occur at home and not in hospital [28], and because causes of death from VA are obtained differently from inpatient death certificates [11]. Nevertheless, the sample for measuring malaria-attributable mortality is limited since malaria was attributed as most probable cause by InterVA-4 software in only 12 of the 86 deaths analyzed, and as second most probable cause in two additional deaths. The performances of the previous version of this software has been suboptimal, especially regarding malaria [29–31]. The current version of InterVA seems to perform better, similar to the assignment of causes of death by physicians [16, 32–34], but all tools for interpretation of VA results are poorly accurate in the identification of death attributable to malaria [34]. In the absence of training dataset for the assignment of causes of death, InterVA is the only automated option or analysing VA data [16]. Because of the small size of the sample, and because vital registration system of Madagascar is not comprehensive, results were not weighted. As far as the authors know, no study about causes of death has ever been conducted across Madagascar. This study, with the limitations cited above, thus provides baseline results for CSMF in the general population of Madagascar.

Finally, this study provides information about factors affecting *Plasmodium* infection that were not collected in MIS. For example, domestic use of insecticides is widespread in Madagascar since 22.8% of the population live in households where insecticides other than IRS are used. This may be one of the factors explaining socio-economic differences in terms of *Plasmodium* infection, together with already documented factors such as access to prophylaxis and treatment, housing quality and nutritional status [21].

Numerous KAP surveys regarding malaria have been conducted, including MIS, but the exposure to IEC messages and analysis of media through which those messages are disseminated have less been investigated [27, 35, 36]. Results show that people belonging to upper SES and the more educated people are more exposed to, or better retain IEC messages about malaria from all media, except information meetings. Remarkably, most media broadcast better in areas of low population density, i.e., rural areas where malaria transmission is the highest, than in high-density areas, i.e., urban areas. As expected, television was an exception but also, more surprisingly, MVU that is designed to reach remote settings [37]. This result suggests that the design of IEC messages should pay more attention to reduce socio-economic inequities. The association between exposure to IEC messages and better coverage of malaria control interventions remain to be investigated, and will be presented later in a separate publication.

This study also presents various shortcomings. First, only RDT results and no thick smear results are available. Since 58.1% (317/546) of slides from individuals with a positive RDT results were found to be negative by microscopy, RDT result was selected for the assessment of *Plasmodium* infections. This high proportion of negative slides can be explained either by a lack of sensitivity of microscopy in the context of low *Plasmodium* transmission and low parasite density in this season, or by a problem in the preparation of slides. This second explanation is more plausible because the agreement between the RDTs used in the present study and microscopy has been demonstrated earlier in Madagascar [38], and because the preparation of the slides were performed in the households of participants, thus often on uneven surfaces. Separate results for non- *P. falciparum* infection were not presented because 99.6% of slides positive by microscopy exhibited *P. falciparum*, and only one slide was found positive for *P. vivax*. It was thus estimated that a positive RDT result was a reasonable proxy for *P. falciparum* infection.

Since clusters were chosen in the vicinity of health facilities belonging to fever sentinel network, the sample cannot be considered as strictly representative of the country. Additionally, numerous absences, refusals and/or impossibilities to investigate places and households (21.0% of *fokontany* and 17.9% of households) were noted, which could introduce a bias in the sampling. These pitfalls were overcome by applying weighting by age, sex, transmission pattern, and population density to the data, as described in Methods. As a consequence, the weighted figures observed were in rather good accordance with previous and subsequent studies (IRS, IPTp, and case management, Table 10). This establishes the proof-of-concept of the methodology used in the present study for the evaluation of nationwide indicators.

Another shortcoming is the collection of data about clinical cases in SHCs that belong to the public sector. The population of clinical cases is thus blinded for consultants in private sector, which may introduce a bias although this proportion was less than 30% in the CSS sample. Additionally, no excessive proportions of under-fives or pregnant women was observed in clinical cases, although they benefit from free care in the public sector. It was thus assumed that, if there is any bias, its importance may be limited.

## Conclusions

The original design of this study provided concurrent results about *Plasmodium* infection, malaria morbidity and mortality attributable to malaria at a nationwide level. Results largely confirm facts described elsewhere, but some deserve to be underlined. First, the variation in LLIN coverage may be largely overestimated if only one season is considered while collecting indicators. Second, when malaria control interventions not only try to reduce burden but also aim to reduce *Plasmodium* transmission, other subgroups than under-fives and pregnant women, or even the entire population, should be considered as targets for control interventions and evaluation surveys. In addition, there was concern that people above the age of five in Madagascar may bear an important burden of malaria in terms of infection, morbidity and mortality. Third, important socio-economic inequalities have been found, in the prevalence of infection as described previously, but also in exposure to IEC messages and in domestic use of insecticides. The authors plead for more attention to these issues while public health policy makers design and implement malaria control interventions or conduct evaluation surveys.

## Electronic supplementary material

Additional file 1: **Wealth component scores and measures across quintiles.** Table displaying wealth component scores and measures across quintiles in the CSS. (XLSX 14 KB)

Additional file 2: **Age distribution of males and females in the cross-sectional survey.** Table displaying crude and weighted numbers and proportions of male, female and total according to age categories. (XLSX 11 KB)

Additional file 3: **Distribution of socio-economic status quintiles in transmission patterns.** Table displaying crude and weighted numbers and proportions of SES quintiles according to transmission patterns. (XLSX 12 KB)

Additional file 4: **Distribution of socio-economic status quintiles and levels of education according to population density.** Table displaying crude and weighted numbers and proportions of SES quintiles and levels of education according to population density. (XLSX 12 KB)

Additional file 5: **Distribution of levels of education according to transmission patterns.** Table displaying crude and weighted numbers and proportions of levels of education according to transmission patterns, among people ≥15 years old. (XLSX 10 KB)

Additional file 6: **Age distribution of parasite rates.** Table displaying weighted parasite rates (PR) in males, females and total according to age categories. (XLSX 10 KB)

Additional file 7: **Distribution of long-lasting insecticidal nets, non-impregnated bed nets and no bed net users, according to definition, transmission pattern and population density.** Table displaying weighted proportions of LLIN users, NIBN users and people not using bed nets, whether users are defined according to use the previous night, or every night during previous three months, according to transmission patterns and population density. (XLSX 10 KB)

Additional file 8: **Sociodemographic characteristics of non-impregnated bed nets users the previous night.** Table displaying weighted numbers and proportions of NIBN users the previous night, according to sociodemographic characteristics. (XLSX 13 KB)

## Abbreviations

ACM: Appropriate case management
ACT: Artemisinin-based combination therapy
CCS: Clinical case study
CHW: Community Health Worker
CSMF: Cause-specific mortality fraction
CSS: Cross-sectional survey
DDT: Dichlorodiphenyltrichloroethane
GPS: Global positioning system
IEC: Information, Education and Communication
IPTp: Intermittent preventive treatment of pregnant women
IRS: Indoor residual spraying
KAP: Knowledge, attitude and practice
LLIN: Long-lasting insecticidal nets
MEDALI: *Mission d’Etude des Déterminants de l’Accès aux Méthodes de Lutte antipaludique et de leur Impact*
MIS: Malaria Indicator Survey
MVU: Mobile video units
NIBN: Non-impregnated bed net
OR: Odds ratio
PR: Parasite rate
RDT: Rapid diagnostic tests
SES: Socio-economic status
SHC: Sentinel health centres
SP: Sulphadoxine-pyrimethamine
VA: Verbal autopsy.
